# Reproduction and signals regulating worker policing under identical hormonal control in social wasps

**DOI:** 10.1038/s41598-020-76084-4

**Published:** 2020-11-04

**Authors:** Cintia Akemi Oi, Robert L. Brown, Rafael Carvalho da Silva, Tom Wenseleers

**Affiliations:** 1 Laboratory of Socioecology and Social Evolution, KU Leuven, Leuven, Belgium; 2 Manaaki Whenua – Landcare Research, Lincoln, New Zealand; 3 Faculdade de Filosofia, Ciências e Letras de Ribeirão Preto, Departamento de Biologia, Universidade de São Paulo – USP, Ribeirão Preto, SP Brazil

**Keywords:** Chemical ecology, Animal behaviour, Zoology

## Abstract

In social Hymenoptera, fertility and fertility signalling are often under identical hormonal control, and it has been suggested that such hormonal pleiotropies can help to maintain signal honesty. In the common wasp *Vespula vulgaris*, for example, fertile queens have much higher juvenile hormone (JH) titers than workers, and JH also controls the production of chemical fertility cues present on the females’ cuticle. To regulate reproductive division of labour, queens use these fertility cues in two distinct ways: as queen pheromones that directly suppress the workers’ reproduction as well as to mark queen eggs and enable the workers to recognize and police eggs laid by other workers. Here, we investigated the hormonal pleiotropy hypothesis by testing if experimental treatment with the JH analogue methoprene could enable the workers to lay eggs that evade policing. In support of this hypothesis, we find that methoprene-treated workers laid more eggs, and that the chemical profiles of their eggs were more queen-like, thereby causing fewer of their eggs to be policed compared to in the control. Overall, our results identify JH as a key regulator of both reproduction and the production of egg marking pheromones that mediate policing behaviour in eusocial wasps.

## Introduction

Juvenile hormone (JH) is known to influence multiple biological processes in insects, including metamorphosis, diapause and reproduction, which makes it a prime example of a pleiotropic hormone^1–3^. This means that JH can regulate different functions in insects like reproductive physiology and behaviour^4^. In social insects, as in solitary ancestors, JH frequently retains a gonadotropic effect enhancing female reproduction^5–7^, but has also acquired secondary functions, like regulating behavioural specialisation and division of labour^3,4,8^ or mediating the production of queen and fertility signals^5,6,9–11^. The dual effect that JH has on reproduction and the production of queen and fertility signals in some species has been suggested to help maintain signal honesty^12^. The honesty of the signal can be kept due to the fact that they represent an index (intrinsically physiological link), due to a handicap (only high quality individuals can produce such a signal) or if align benefits to the sender and the receiver^13^. Queen pheromones, specific hydrocarbons in the cuticle of the queen, align the interests of the queen and workers and can be used as honest indexes of fertility^11,14^. The hormonal pleiotropy hypothesis received support in the common wasp *Vespula vulgaris,* where fertile queens had very high JH titers compared to workers and where queens produced specific long-chained hydrocarbons on their cuticle, correlating JH titers and queen pheromones^12,14^. In addition, workers that were experimentally treated with the JH analogue methoprene acquired more queen-like cuticular profiles^12^. These results, correlational evidence and causal relationship together, demonstrated that reproduction and the production of fertility-linked signals were under identical hormonal control.

In social Hymenoptera, fertility signals are known to regulate reproduction in several distinct ways. In ants, bees and wasps, queens have been shown to produce specific cuticular hydrocarbons on their cuticle, which act as fertility signals and suppress the daughter workers from reproducing^14–16^. In the common wasp, for example, the long-chain hydrocarbons C27, C29 and 3-MeC29, were all overproduced on the cuticle of fertile queens compared to the cuticle of workers and administration of synthetic versions of these compounds inhibited worker ovary activation^14^. In that same species, 3-MeC29 was also shown to be used by the queen to mark her eggs and enable the workers to recognize and police eggs laid by other workers^17^. Such policing is a widespread conflict-reducing mechanism in insect societies, and involves either the queen or other workers aggressing reproductive workers or removing worker-laid eggs to increase the genetic relatedness to the male offspring that is reared or increase colony productivity^18,19^. The pheromones used in policing behaviour have also been investigated in a few other social insects. In the ant *Aphaenogaster cockerelli*, the linear alkane C25 was found to be more abundant on the cuticle of reproductive “cheater” workers, and experimental application of this hydrocarbon onto the cuticle of non-reproductive workers caused workers to be physically policed^20^. In the ant *Camponotus floridanus*, queen-laid and worker-laid eggs have been shown to display consistent differences in their hydrocarbon profiles and application of queen extracts onto worker-laid eggs resulted in decreased egg policing^21^. Likewise, the queen-characteristic hydrocarbon 3,11-diMeC27 has been implicated as an egg-marking pheromone in the ant *Pachycondyla inversa*^22^. In the monogynous ant *Dinoponera quadriceps* alpha females (= queens) overproduce one unsaturated hydrocarbon (9-C31:1) when compared to other nest members, in the same way their eggs are covered with higher proportions of the same compound, which is likely to enhance policing^23^. In the honeybee, where worker policing was first discovered^24^, the identity of the egg marking pheromone remains elusive, with early studies indicated that egg-marking pheromones were esters derived from the queen’s Dufour’s gland^25–27^, but later evidence suggested others as yet unknown bioactive compounds^28–32^.

Given that worker-laid eggs are so effectively policed, rare workers might be able to gain direct fitness benefits if they could lay eggs that evade policing, e.g. by laying eggs that chemically mimic the ones laid by the queen^19^. Although there is policing, workers would nonetheless benefit by escaping and cheating, and an interesting question is: what mechanisms could help to prevent such cheating^19^? In some anarchistic lineages of honeybees as well as in parasitic Cape honeybees, workers have been documented to cheat by laying eggs that can evade policing^33,34^, apparently by coating their eggs with pheromones that are normally characteristic for queens^31,35,36^. Building on the hormonal pleiotropy hypothesis^12^, we here propose that such cheating would be more difficult in species where fertility and the production of fertility signals is under joint hormonal control, as this would make it harder for workers to lie about their true fertility and adequately mimic a queen signal.

In the present study, we test this hypothesis in the common wasp *V. vulgaris*, which is a model species in studies on worker policing^17,37,38^. In particular, we carry out hormonal manipulations, whereby we topically treat workers with the JH analogue methoprene, in which previously it was shown to affect the production of cuticular fertility cues^12^ and test if this would affect worker egg laying rates and also cause the workers to lay eggs with more queen-like chemical profiles and reduced rates of policing.

## Results

**Figure 1:**
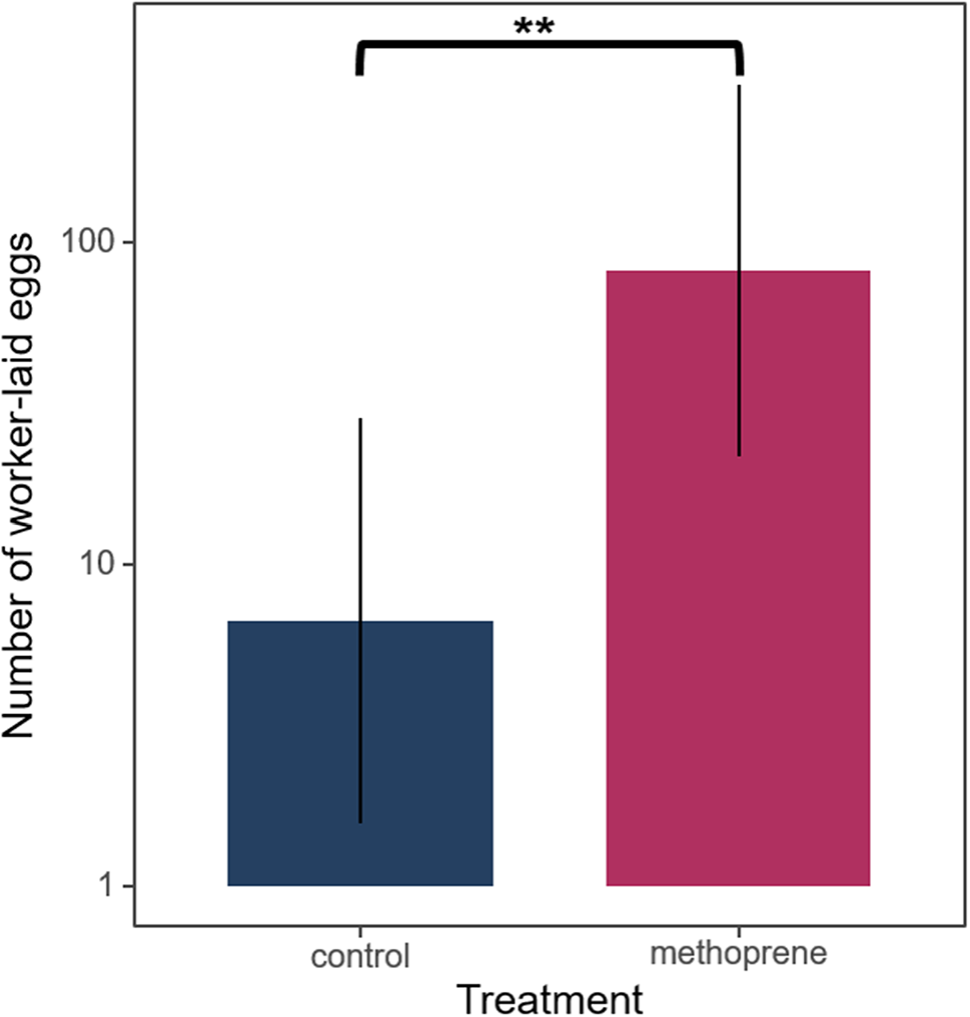
JH has a gonadotropic effect in *V. vulgaris* workers. This is shown in the barplot by the fact that the total number of eggs laid by workers treated with the JH analogue methoprene over a period of one week were significantly higher than in the acetone solvent-treated control workers (Poisson GLMM, z = 3.171, *p* = 0.0015**). Error bars indicate 95% confidence intervals.

Previously, Oliveira et al. (2017)^12^ found an effect of fertility for queens and not for workers, although with two weeks, all the treated workers showed the maximum of ovary activation (level V). Using egg-laying rates as a proxy of fertility, we observed that workers treated with the JH analogue methoprene laid significantly more eggs over a period of one week compared to the acetone solvent-treated control workers (Poisson GLMM, z = 3.171, *p* < 0.01**) (Fig. 1, Table 1a). Considering that worker fertility is under juvenile hormone control, this result was expected.

**Table 1:** (a) The total number of eggs laid by workers over a one week period were compared across the methoprene and control treatments using a Poisson generalized linear mixed model (GLMM) in which colony was coded as a random intercept and an observation-level random effect was included to take into account overdispersion. (b) The proportion of eggs laid by methoprene and control workers that were policed within a period of 24 h were compared using a binomial GLMM. In this analysis, colony was included as a random intercept and an observation-level random effect was included to take into account overdispersion. Coefficients, standard errors, z-values and *p*-values of the fixed treatment effect are shown.

	Estimate	Std. Error	z value	*p*-value	
**(a) Number of eggs laid by workers**					
(Intercept)	1.899	0.737	2.577	0.009	**
Methoprene	2.505	0.790	3.171	0.002	**
**(b) Proportion of eggs policed**					
(Intercept)	− 0.943	0.417	− 2.265	0.023	*
Methoprene	− 1.109	0.265	− 4.185	0.000	***

**Figure 2:**
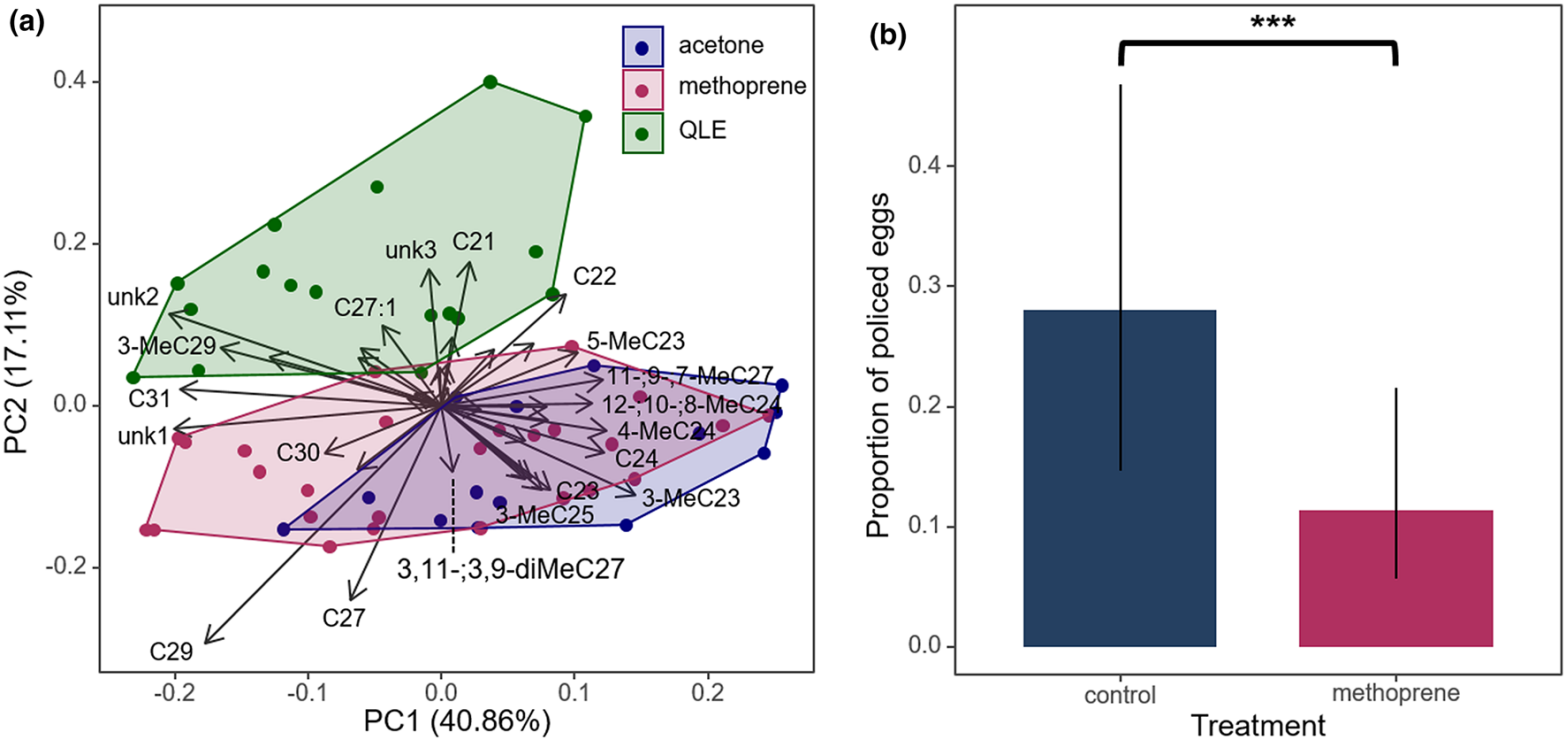
Workers treated with the JH analogue methoprene laid eggs that were more queen-like and were policed less. (**a**) PCA analysis of the chemical surfaces of the eggs, based on centered log-ratio transformed peak areas, shows that methoprene-treated workers laid eggs that slightly shifted towards the profile typical of queen-laid eggs. (**b**) Methoprene-treated workers laid eggs that were more queen-like and consequently were also less likely to be policed within 24 h than those laid by control workers (binomial GLMM, z = -4.185, *p* < 0.001***). Error bars indicate 95% confidence intervals.

**Figure 3:**
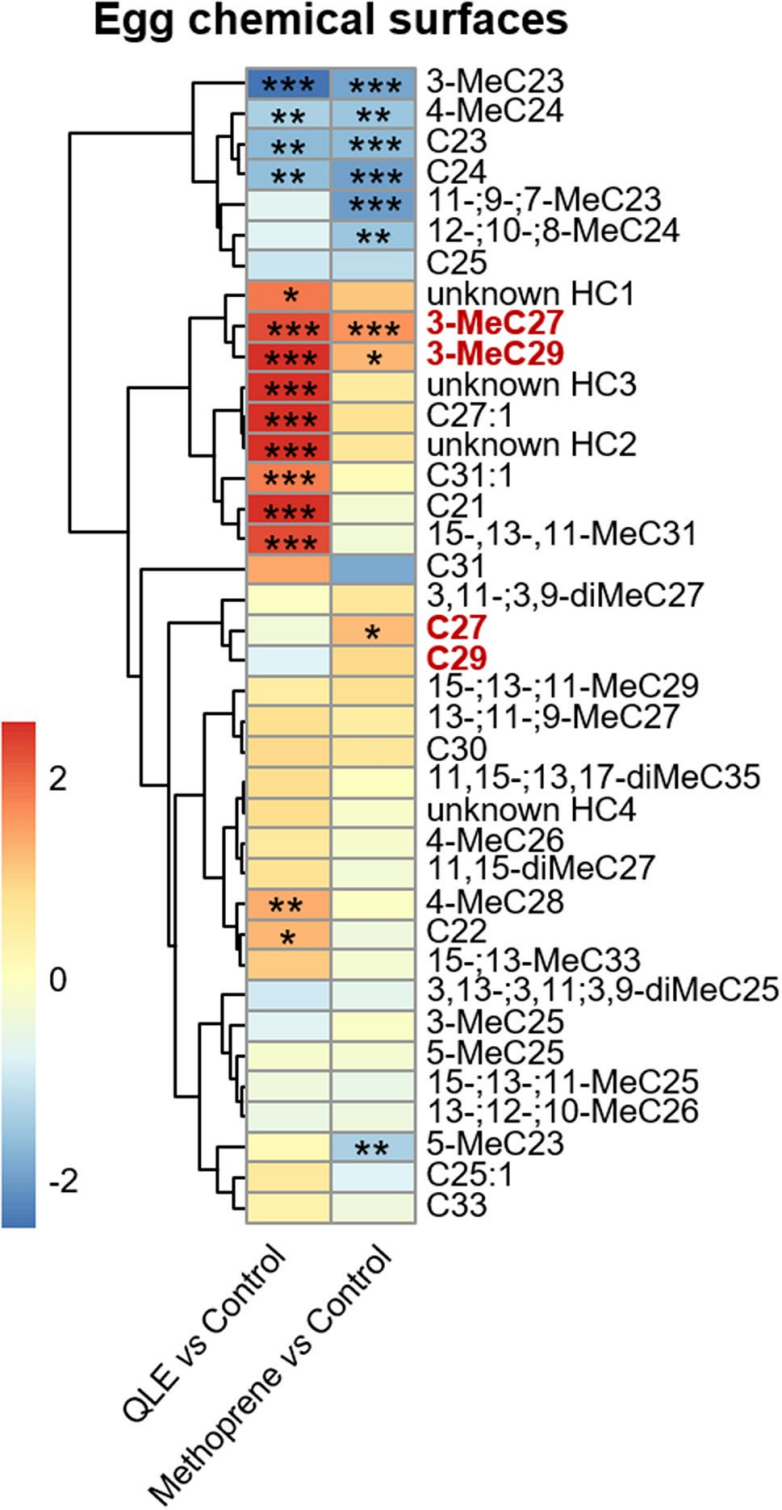
Heatmap showing differences in the chemical profiles of queen-laid eggs (QLE) and worker-laid eggs in the control groups (control) and how treatment with the JH analogue methoprene caused workers to lay eggs that became more queen-like. Colours indicate the mean fold difference in relative abundance of each hydrocarbon on the surface of queen-laid eggs and eggs laid by methoprene-treated workers compared to the eggs laid by the acetone solvent-treated control workers. Compounds were clustered based on a UPGMA hierarchical clustering using Euclidean distance as the distance metric. Asterisks indicate the FDR-corrected significances of individual contrasts for cases where the absolute fold difference was greater than 1.5 based on ANOVA. Highlighted red the compounds previously identified queen pheromones (C27, C29 and 3-MeC29)^14^ and queen egg marking pheromones (3-MeC27 and 3-MeC29)^17^.

**Figure 4:**
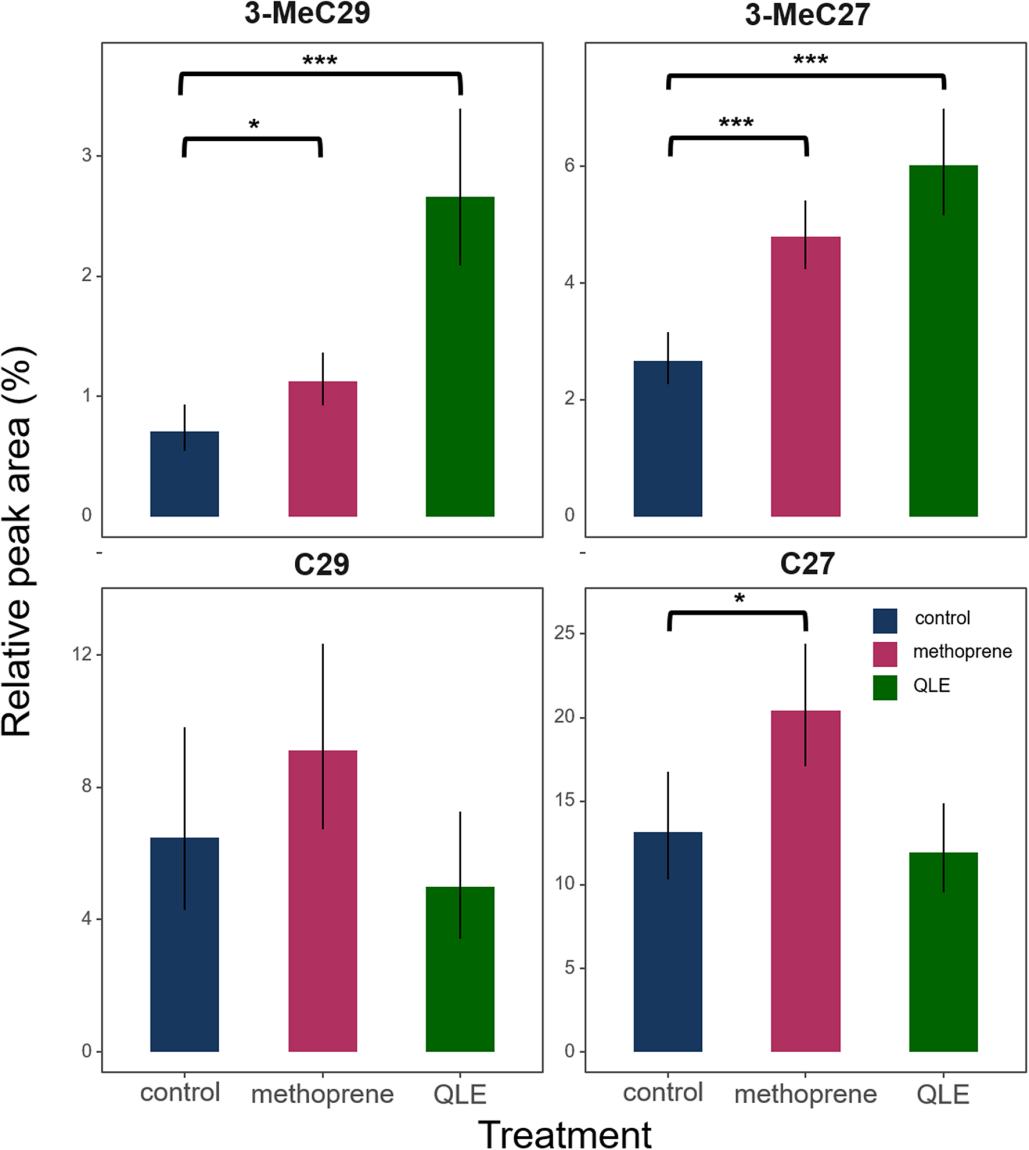
Bar plots of the relative peak areas of compounds present on the egg surface that were previously identified as egg-marking pheromones (3-MeC27 and 3-MeC29^17^) or as sterility-inducing queen pheromones (C29, C27 and 3-MeC29^14^) in the common wasp *V. vulgaris* in our different groups (control, methoprene and queen-laid eggs QLE). Asterisks indicate FDR-corrected significances in the log2-transformed relative peak areas compared to the acetone solvent-treated control group. Error bars indicate 95% confidence intervals of linear modes fit on log2 relative peak areas. The two methyl-branched hydrocarbons 3-MeC29 and 3-MeC27 were significantly more abundant on queen-laid eggs (QLE)^17^ and became significantly more abundant on worker-laid eggs if workers were treated with the JH analogue methoprene. The linear alkanes C27 and C29, by contrast, were not significantly more abundant on queen-laid eggs (QLE) than control worker-laid eggs, but previous research has shown these compounds to be characteristic for the queen cuticle and act as worker-sterility inducing queen pheromones^14^. One of these compounds, C27, also became more abundant on worker-laid eggs following methoprene treatment.

**Figure 5:**
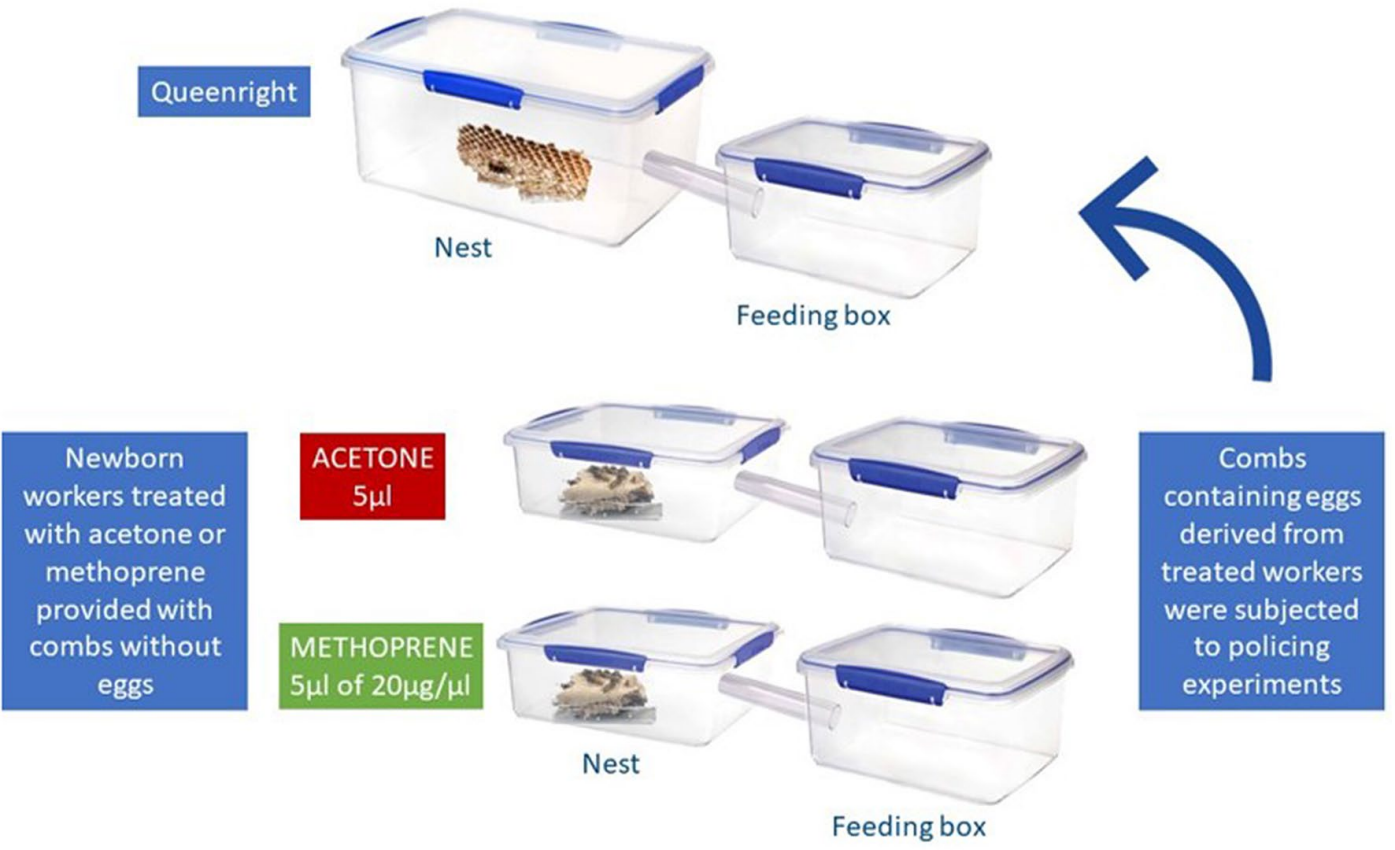
Experimental setup used to test for the effect of treatment with the JH analogue methoprene on worker fertility and rates of egg policing in the common wasp *V. vulgaris*.

Furthermore, our hypothesis that the production of the egg-marking signal would also be under joint JH control was supported by the fact that eggs laid by workers treated with methoprene became more queen-like, as demonstrated by a PCA (Fig. 2a) and by the significantly increased production of compounds that are characteristic for either queen-laid eggs (e.g. 3-MeC29 and 3-MeC27, Fig. 3^17^) or the queen’s cuticle (C27,^12,14^) (see Figs. 3 and 4 and Table S1). In line with this, we found that the queen-like eggs laid by the methoprene-treated workers were less likely to be policed within a period of 24 h than those laid by the control workers (binomial GLMM, z = -4.185, *p* < 0.001***, Fig. 2b, Table 1b). Interestingly, one of the compounds that was overproduced in the methoprene-treated workers, 3-MeC29 (Fig. 4), was previously identified to act both as a worker-sterility inducing compound and as a queen egg-marking pheromone in the common wasp^14,17^, while C27 has previously been shown to act as one of the main sterility-inducing queen pheromones in this species^14^, being characteristic for the queen’s cuticle, though not for queen-laid eggs (Fig. 5^14,17^). In total, we were able to integrate 38 peaks in the egg chemical profiles, and these comprised mostly from hydrocarbons, of which 34 were successfully identified as linear alkanes (n = 10), monomethylalkanes (n = 15), dimethylalkanes (n = 4) and alkenes (n = 3) (Table S1). The results shown in heatmap (Fig. 3), showed 3 compounds (3-MeC27, 3-MeC29 and C27) significantly overproduced by methoprene treatment compared to the control and several compounds (3-MeC27, 3-MeC29, C27:1, C31:1, C21, 4-MeC28, C22 and 3 unidentified compounds) overproduced both on QLE and methoprene-treated workers, and others that were queen-characteristic and overproduced in methoprene treated workers but not characteristic for QLEs (C27 and C29).

## Discussion

In the common wasp *V. vulgaris*, reproduction and production of fertility cues on the cuticle of females have been shown to be under shared JH control^12^. Such hormonal pleiotropy was suggested to be a mechanism that would help maintain the honesty of the produced fertility signals^12^. In the present study, we find additional support for the hormonal pleiotropy hypothesis, and show that fertility and the production of egg-marking signals used to regulate worker policing are also both under JH control. In particular, we found that treatment of common wasp workers with the JH analogue methoprene caused workers to lay more eggs, thereby demonstrating JH to have a gonadotropic effect, and we further show that the methoprene treatment caused workers to lay more queen-like eggs that were policed less compared to those laid by control workers. This implies that in the common wasp, not only the production of cuticular queen pheromones but also that of queen egg-marking signals are under hormonal control.

The gonadotropic effect of JH observed in common wasp workers in this study is interesting, and contrasts with a previous study that failed to find any effect of methoprene treatment on worker ovary development in that species^12^. That study suggested that a gonadotropic effect in social wasps was limited to queens in advanced eusocial species (based on the observed high JH titer in queens^12^), or foundresses in primitively eusocial species lacking morphologically specialized castes (e.g. in^7,39,40^). In the worker caste of many social wasps, by contrast, it was proposed that JH had acquired novel functions, unrelated to fertility, as appears to be the case in some primitively eusocial Polistinae wasps, swarm-founding eusocial Polistinae wasps, in the leaf-cutting ant *Acromyrmex octospinosus* and the advanced eusocial honeybee, where JH regulates worker polyethism and the transition from nursing to foraging^4,41–44^. Whether JH also has effects on worker polyethism in the common wasp currently remains unknown and should be investigated in further studies. A likely explanation for our difference in conclusion is the fact that Oliveira et al. (2017)^12^ measured worker ovary development, while in the present study we directly measured worker egg-laying, which is arguably a more direct measure of fertility. In addition, Oliveira et al. (2017)^12^ left workers to activate their ovaries for 14 days, while this was only 7 days in the present study. Hence, it is possible that the methoprene treatment mainly induced workers to commence egg-laying sooner, and that this effect was not detected in the earlier trial with a longer duration^12^. The gonadotropic effect of JH could have come about in one of two ways: by increasing the fecundity of reproductive workers, or by increasing the probability that workers would become reproductive. Next studies could test if some workers were more prone to cheat and avoid hormonal control with additional genetic or genomic work, e.g. to look at worker patrilines differences in worker ovary activation.

Our key result that methoprene treatment of workers causes such workers to lay eggs with a more queen-like chemistry ties in with earlier results that JH plays a key role in regulating the production of cuticular queen pheromones and fertility cues in many species of social insects^5,12,45^. In fact, JH-induced changes in cuticular profiles in combination with JH-induced gonadotropic effects have also been documented in several solitary insect species^46–53^. In social wasps, and outside Vespinae wasps, JH-mediated production of fertility signals has also been demonstrated in several polistine wasps, including *Synoeca surinama* and *Belanogaster longitarsus*^5,6^. Also recently, gonadotropic effects of JH were documented in *Polistes fuscatus*^54^ and *P. chinensis*^55^*.* In the common wasp *V. vulgaris*, known fertility signals comprise both worker sterility-inducing hydrocarbons produced on the queen’s cuticle, including the linear alkanes C29 and C27 and the methyl-branched hydrocarbon 3-MeC29, as well as hydrocarbons that are more abundant on the surface of queen-laid eggs, including 3-MeC29 and 3-MeC27, of which the former was shown to double up as a sterility-inducing queen pheromone and a queen egg-marking pheromone^14^. Interestingly, methoprene treatment of workers in our experiments caused workers to lay eggs with increased abundance of both sets of compounds, i.e. compounds that are both characteristic for queen-laid eggs (3-MeC29 and 3-MeC27)^17^ and for the queen’s cuticle, with the latter including several bioactive sterility-inducing queen pheromones (C27 and 3-MeC29)^14^. The increased production of the known queen egg-marking pheromone 3-MeC29 and the overall more queen-like chemistry of the eggs laid by the methoprene-treated workers likely explains the observed reduced rate of egg policing. These results show that JH, therefore, does not only regulate fertility and the production of fertility-linked queen signals, but also the queen egg-marking signals that workers use to discriminate between queen-laid and worker-laid eggs during worker policing^17^.

If by increasing their JH titer, workers could lay eggs that could evade policing, the question arises if such cheating would be possible in nature. An interesting example of such cheating occurs in rare lineages of anarchistic honeybee^33,34^ (reviewed in^56^), where workers can reproduce in the presence of the queen, upregulate the queen pheromone 9-HDA in their mandibular glands and lay eggs with high levels of queen-like Dufour’s gland esters, presumably helping them and their eggs to evade policing^31,35,36^. Likewise, the clonally reproducing Cape bee, *Apis mellifera capensis*, has been shown to mimic the queen’s Dufour gland esters, queen mandibular gland secretions and tergal gland pheromones, thereby causing workers to be treated as ‘false queens’ and achieve reproductive dominance in *Apis mellifera scutellata* host colonies^27,34,56^. Hormonal pleiotropy, and the fact that JH appears to regulate both fertility and the production of the queen egg-marking signals, would be expected to make such cheating harder in the common wasp. In principle, however, there could also be selection for both traits to become decoupled, and in that case cheating might still be possible^57^ . One factor that could select against worker cheating is that if workers would coat their eggs with a more queen-like profile they could also give away their fertility status via the correlated expression of queen-like cuticular signals and that this could induce other workers to police them via targeted aggression. At present, it is not known if common wasp workers are also policed via aggression, but policing via aggression has been documented in a number of ants, bees and wasps^20,58–61^. An alternative reason that workers might not be able to cheat is if circulating levels of JH would be strictly tied to body size and the size of the corpora allata, where it is biosynthesized, as the JH titer would then become an uncheatable index of fertility. This hypothesis is at present speculative and requires further testing.

From an evolutionary perspective, advanced eusociality led to the emergence of a queen-worker reproductive division of labour coupled to highly efficient queen signalling systems. In support of the hormonal pleiotropy hypothesis^12^, we showed that both systems are tightly linked and are under joint hormonal control. Furthermore, we showed that the queen signals that are being regulated by JH also include the queen pheromones involved in signalling egg maternity, which allow the workers to selectively police worker-laid eggs. Future studies could be targeted to determining why fertility and the production of fertility signals could not become decoupled^57^, as one would expect workers to gain large fitness benefits if they could evade egg policing by mimicking the queen signals. In other words, the question arises under what selective regimes the pleiotropy required to limit or prevent such cheating could evolve. Both theoretical modelling and molecular studies, aimed at investigating the molecular pathways by which JH affects fertility and fertility signalling, could shed some light on this question. Finally, it would be desirable to test in what other social insect species hormonal pleiotropies apply and to what extent this may help to maintain the honesty of the queen signals that are produced.

## Material and methods

Thirteen colonies of *V. vulgaris* were collected near Springfield (New Zealand) in February 2019. They were transported to the animal facilities of Manaaki Whenua—Landcare Research in Lincoln, where they were shortly anaesthetized with carbon dioxide and divided in closed artificial plastic nests. The combs were kept in a separate box from the workers and the queen. For the thirteen colonies, newborn workers were collected after two days and divided into two groups of 25 workers each, which were treated with 5 µl per worker of 20 µg/µl of methoprene diluted in acetone or 5 µl of acetone only, to serve as a control. The compounds were applied topically on the abdomen. The amount of methoprene used was based on a previous study performed by Oliveira et al. (2017)^12^. Treated workers were kept in separated small experimental boxes, in which a comb without eggs was offered. Water, sugar syrup and protein were offered ad libitum in the feeding box. A third larger queenright part of each colony was kept serving as a host colony for the policing assays. This queenright part contained two big combs, the queen and ca.100 workers, which were placed in an experimental nest box connected to a small foraging box. Experimental boxes were made using plastic containers (Sistema) of 10 L (queenright colony part), 5 L (foraging box) and 3 L (worker comb) connected through a vinyl tube of 3 cm in diameter (Fig. 5). Ventilation holes were provided on the side and in the top of the nest boxes. A thin wire was used to support the combs in the nest box.

Treated workers were kept for 7 days to obtain worker-laid eggs. After a week, all the worker laid eggs were counted and the specific positions of all eggs were registered in a cell map. At the start of each policing trial, the combs containing worker-laid eggs from the methoprene-treated or acetone-treated worker groups were then introduced side by side into the respective queenright colony part. After 24 h, the combs were then removed, the remaining eggs counted, and 1 to 3 eggs per colony were collected for chemical analysis. To check for possible effects of the methoprene treatment of worker fertility, we compared the total number of worker-laid eggs using a Poisson generalized linear mixed model (GLMM), whereby treatment was coded as a fixed factor, colony as a random factor and an additional observation-level random effect was included to take into account overdispersion. The proportion of eggs that were policed were compared using a binomial GLMM, whereby treatment was coded as a fixed factor, colony as a random factor and an additional observation-level random effect was included to consider overdispersion. An alternative model was also fitted in which the number of newly-laid eggs (presumably laid by the queen) were included as an additional covariate, but this model had a lower Akaike Information Criterion, and so was less parsimonious and we left this model out. All statistical analyses were performed using R version 4.0.2.

To compare the chemical profiles of eggs laid by control and methoprene-treated workers, we collected 1 to 3 worker-laid eggs from both treatments and 1 to 3 queen-laid eggs from each of the 13 colonies (total n = 14, 25 and 17 for the control treatment, methoprene treatment and queen-laid eggs). The eggs were then chemically extracted by immersing them in 50 µl of hexane for 1 min. These extracts were then transferred into glass inserts and dried down samples were shipped to the Laboratory of Socioecology and Social Evolution (KU Leuven, Belgium) for GC/MS analysis. After resuspending the samples in 20 µl of hexane (HPLC grade), GC runs were performed using a Thermo Fisher Trace 1300 / ISQ GC/MS equipped with a Restek MXT-5 column (30 m, 0.25 mm and 0.25 µm film thickness). To this end, 1 µl of each sample was injected using splitless injection at 320 °C and a final pressure of 75 kPa. Initially the temperature was held at 40° C for 2 min, then increased to 120° C with an increase of 20° C/min, followed by an increase of 10° C/min until 200 °C, then 7° C/min to reach 250° C and a last increase of 5° C to 350 °C/min which was held for 4 min. The helium carrier gas had a constant flow rate of 0.9 mL/min. Mass spectrometry was performed with electron impact (EI) at 70 eV. Alkane ladders (n-C7 to n-C40, Supelco) were run in the same program at two different concentrations (0.01 µg/µl and 0.005 µg/µl) to be able to calculate cubic spline-interpolated retention indexes^62^. Peak integration was performed by integrating over total ion chromatograms using in-house developed software in R v.4.0.2 (script available upon request from the authors). Hydrocarbon peaks were identified on the basis of expected mass spectrometric fragmentation patterns^63,64^, expected retention indices (available online in the NIST Chemistry Webbook,^65^) and comparison with earlier analyses^12,17^. A principal component analysis (PCA) on centered log-ratio transformed peak areas (appropriate for compositional data,^66^) was performed using R’s prcomp function of the stats package. Log transformed relative peak areas were also compared using Anova analyses to check for univariate differences among treatment groups. In these analyses, significance levels were FDR corrected for multiple testing. All statistical analyses were performed using R version 4.0.2^67^.

## Supplementary information

Supplementary Information

## Data Availability

Raw tables of number of eggs and raw peak areas will be included in online repository.
