# Supplementary material for: Reproduction and signals regulating worker policing under identical hormonal control in social wasps
Source: Sci Rep. 2020 Nov 4;10:18971. doi: 10.1038/s41598-020-76084-4 (PMC7643062; doi:10.1038/s41598-020-76084-4)
Supplement: Supplementary file 1 — Supplementary Information [file 41598_2020_76084_MOESM1_ESM.docx]

**Reproduction and signals regulating worker policing under identical hormonal control in social wasps**

Authors: Cintia Akemi Oi^1*^, Robert L. Brown^2^, Rafael Carvalho da Silva^3^, and Tom Wenseleers^1^

^1^ Laboratory of Socioecology and Social Evolution, KU Leuven, Leuven, Belgium

^2^ Manaaki Whenua – Landcare Research, Lincoln, New Zealand

^3^ Universidade de São Paulo – USP/ Faculdade de Filosofia, Ciências e Letras de Ribeirão Preto, Departamento de Biologia, SP, Ribeirão Preto, Brazil

Contact email: cintiaakemioi@gmail.com

Supplemental

Table S1: Pairwise comparisons on log transformed relative peak areas among treatment groups. In these analyses, significance levels were FDR corrected for multiple testing per compound between control vs methoprene and control vs queen-laid eggs.

|  | **compound** | **contrast** | **estimate** | **SE** | **df** | **t.ratio** | **p.value** | **padj** | **folddiff** |
| --- | --- | --- | --- | --- | --- | --- | --- | --- | --- |
| 1 | 11-;9-;7-MeC23 | control - methoprene | 0.97 | 0.21 | 54 | 4.53 | 0.00 | 0.00 | 1.96 |
| 2 | 11-;9-;7-MeC23 | control - QLE | 0.34 | 0.23 | 54 | 1.45 | 0.15 | 1.00 | 1.26 |
| 4 | 11,15-;13,17-diMeC35 | control - methoprene | -0.02 | 0.21 | 54 | -0.08 | 0.93 | 1.00 | -1.01 |
| 5 | 11,15-;13,17-diMeC35 | control - QLE | -0.46 | 0.23 | 54 | -2.03 | 0.05 | 1.00 | -1.38 |
| 7 | 11,15-diMeC27 | control - methoprene | 0.13 | 0.19 | 54 | 0.67 | 0.50 | 1.00 | 1.09 |
| 8 | 11,15-diMeC27 | control - QLE | -0.40 | 0.21 | 54 | -1.92 | 0.06 | 1.00 | -1.32 |
| 10 | 12-;10-;8-MeC24 | control - methoprene | 0.72 | 0.20 | 54 | 3.63 | 0.00 | 0.00 | 1.65 |
| 11 | 12-;10-;8-MeC24 | control - QLE | 0.34 | 0.22 | 54 | 1.59 | 0.12 | 1.00 | 1.27 |
| 13 | 13-;11-;9-MeC27 | control - methoprene | -0.27 | 0.17 | 54 | -1.56 | 0.12 | 1.00 | -1.21 |
| 14 | 13-;11-;9-MeC27 | control - QLE | -0.43 | 0.19 | 54 | -2.27 | 0.03 | 1.00 | -1.35 |
| 16 | 13-;12-;10-MeC26 | control - methoprene | 0.20 | 0.18 | 54 | 1.14 | 0.26 | 1.00 | 1.15 |
| 17 | 13-;12-;10-MeC26 | control - QLE | 0.22 | 0.19 | 54 | 1.18 | 0.24 | 1.00 | 1.17 |
| 19 | 15-,13-,11-MeC31 | control - methoprene | 0.12 | 0.26 | 54 | 0.46 | 0.65 | 1.00 | 1.09 |
| 20 | 15-,13-,11-MeC31 | control - QLE | -1.17 | 0.28 | 54 | -4.15 | 0.00 | 0.00 | -2.25 |
| 22 | 15-;13-;11-MeC25 | control - methoprene | 0.25 | 0.19 | 54 | 1.33 | 0.19 | 1.00 | 1.19 |
| 23 | 15-;13-;11-MeC25 | control - QLE | 0.17 | 0.20 | 54 | 0.82 | 0.42 | 1.00 | 1.12 |
| 25 | 15-;13-;11-MeC29 | control - methoprene | -0.42 | 0.17 | 54 | -2.50 | 0.02 | 1.00 | -1.34 |
| 26 | 15-;13-;11-MeC29 | control - QLE | -0.28 | 0.18 | 54 | -1.57 | 0.12 | 1.00 | -1.22 |
| 28 | 15-;13-MeC33 | control - methoprene | 0.11 | 0.19 | 54 | 0.56 | 0.57 | 1.00 | 1.08 |
| 29 | 15-;13-MeC33 | control - QLE | -0.57 | 0.21 | 54 | -2.70 | 0.01 | 1.00 | -1.48 |
| 31 | 3-MeC23 | control - methoprene | 0.93 | 0.20 | 54 | 4.64 | 0.00 | 0.00 | 1.91 |
| 32 | 3-MeC23 | control - QLE | 1.22 | 0.22 | 54 | 5.57 | 0.00 | 0.00 | 2.33 |
| 34 | 3-MeC25 | control - methoprene | 0.03 | 0.17 | 54 | 0.16 | 0.87 | 1.00 | 1.02 |
| 35 | 3-MeC25 | control - QLE | 0.35 | 0.19 | 54 | 1.84 | 0.07 | 1.00 | 1.27 |
| 37 | 3-MeC27 | control - methoprene | -0.84 | 0.15 | 54 | -5.66 | 0.00 | 0.00 | -1.79 |
| 38 | 3-MeC27 | control - QLE | -1.17 | 0.16 | 54 | -7.22 | 0.00 | 0.00 | -2.25 |
| 40 | 3-MeC29 | control - methoprene | -0.66 | 0.24 | 54 | -2.75 | 0.01 | 0.02 | -1.58 |
| 41 | 3-MeC29 | control - QLE | -1.91 | 0.26 | 54 | -7.30 | 0.00 | 0.00 | -3.75 |
| 43 | 3,11-;3,9-diMeC27 | control - methoprene | -0.35 | 0.08 | 54 | -4.37 | 0.00 | 1.00 | -1.27 |
| 44 | 3,11-;3,9-diMeC27 | control - QLE | 0.00 | 0.09 | 54 | -0.04 | 0.97 | 1.00 | -1.00 |
| 46 | 3,13-;3,11;3,9-diMeC25 | control - methoprene | 0.30 | 0.14 | 54 | 2.18 | 0.03 | 1.00 | 1.23 |
| 47 | 3,13-;3,11;3,9-diMeC25 | control - QLE | 0.45 | 0.15 | 54 | 3.05 | 0.00 | 1.00 | 1.37 |
| 49 | 4-MeC24 | control - methoprene | 0.74 | 0.19 | 54 | 3.95 | 0.00 | 0.00 | 1.67 |
| 50 | 4-MeC24 | control - QLE | 0.65 | 0.20 | 54 | 3.22 | 0.00 | 0.01 | 1.57 |
| 52 | 4-MeC26 | control - methoprene | 0.07 | 0.16 | 54 | 0.45 | 0.65 | 1.00 | 1.05 |
| 53 | 4-MeC26 | control - QLE | -0.33 | 0.17 | 54 | -1.88 | 0.07 | 1.00 | -1.25 |
| 55 | 4-MeC28 | control - methoprene | 0.01 | 0.18 | 54 | 0.04 | 0.96 | 1.00 | 1.01 |
| 56 | 4-MeC28 | control - QLE | -0.71 | 0.19 | 54 | -3.67 | 0.00 | 0.00 | -1.64 |
| 58 | 5-MeC23 | control - methoprene | 0.66 | 0.19 | 54 | 3.40 | 0.00 | 0.00 | 1.58 |
| 59 | 5-MeC23 | control - QLE | -0.11 | 0.21 | 54 | -0.51 | 0.61 | 1.00 | -1.08 |
| 61 | 5-MeC25 | control - methoprene | 0.10 | 0.19 | 54 | 0.54 | 0.59 | 1.00 | 1.07 |
| 62 | 5-MeC25 | control - QLE | 0.08 | 0.21 | 54 | 0.38 | 0.70 | 1.00 | 1.06 |
| 64 | C21 | control - methoprene | 0.10 | 0.27 | 54 | 0.37 | 0.71 | 1.00 | 1.07 |
| 65 | C21 | control - QLE | -1.30 | 0.29 | 54 | -4.41 | 0.00 | 0.00 | -2.46 |
| 67 | C22 | control - methoprene | 0.17 | 0.24 | 54 | 0.71 | 0.48 | 1.00 | 1.13 |
| 68 | C22 | control - QLE | -0.66 | 0.27 | 54 | -2.48 | 0.02 | 0.04 | -1.58 |
| 70 | C23 | control - methoprene | 0.82 | 0.19 | 54 | 4.31 | 0.00 | 0.00 | 1.76 |
| 71 | C23 | control - QLE | 0.81 | 0.21 | 54 | 3.90 | 0.00 | 0.00 | 1.75 |
| 73 | C24 | control - methoprene | 0.95 | 0.18 | 54 | 5.13 | 0.00 | 0.00 | 1.93 |
| 74 | C24 | control - QLE | 0.79 | 0.20 | 54 | 3.92 | 0.00 | 0.00 | 1.73 |
| 76 | C25 | control - methoprene | 0.56 | 0.15 | 54 | 3.84 | 0.00 | 1.00 | 1.47 |
| 77 | C25 | control - QLE | 0.48 | 0.16 | 54 | 3.04 | 0.00 | 1.00 | 1.40 |
| 79 | C25:1 | control - methoprene | 0.37 | 0.20 | 54 | 1.86 | 0.07 | 1.00 | 1.30 |
| 80 | C25:1 | control - QLE | -0.33 | 0.22 | 54 | -1.51 | 0.14 | 1.00 | -1.26 |
| 82 | C27 | control - methoprene | -0.64 | 0.22 | 54 | -2.94 | 0.00 | 0.01 | -1.55 |
| 83 | C27 | control - QLE | 0.14 | 0.24 | 54 | 0.59 | 0.55 | 1.00 | 1.10 |
| 85 | C27:1 | control - methoprene | -0.40 | 0.21 | 54 | -1.89 | 0.06 | 1.00 | -1.32 |
| 86 | C27:1 | control - QLE | -1.31 | 0.23 | 54 | -5.66 | 0.00 | 0.00 | -2.47 |
| 88 | C29 | control - methoprene | -0.49 | 0.37 | 54 | -1.33 | 0.19 | 1.00 | -1.40 |
| 89 | C29 | control - QLE | 0.38 | 0.40 | 54 | 0.94 | 0.35 | 1.00 | 1.30 |
| 91 | C30 | control - methoprene | -0.34 | 0.16 | 54 | -2.20 | 0.03 | 1.00 | -1.27 |
| 92 | C30 | control - QLE | -0.48 | 0.17 | 54 | -2.81 | 0.01 | 1.00 | -1.40 |
| 94 | C31 | control - methoprene | 0.91 | 0.39 | 54 | 2.32 | 0.02 | 0.06 | 1.88 |
| 95 | C31 | control - QLE | -0.72 | 0.43 | 54 | -1.69 | 0.10 | 0.16 | -1.65 |
| 97 | C31:1 | control - methoprene | -0.09 | 0.18 | 54 | -0.48 | 0.63 | 1.00 | -1.06 |
| 98 | C31:1 | control - QLE | -0.94 | 0.20 | 54 | -4.82 | 0.00 | 0.00 | -1.92 |
| 100 | C33 | control - methoprene | 0.18 | 0.17 | 54 | 1.06 | 0.29 | 1.00 | 1.14 |
| 101 | C33 | control - QLE | -0.21 | 0.19 | 54 | -1.14 | 0.26 | 1.00 | -1.16 |
| 103 | unknown HC1 | control - methoprene | -0.59 | 0.35 | 54 | -1.71 | 0.09 | 0.16 | -1.51 |
| 104 | unknown HC1 | control - QLE | -0.95 | 0.38 | 54 | -2.54 | 0.01 | 0.04 | -1.94 |
| 106 | unknown HC2 | control - methoprene | -0.36 | 0.29 | 54 | -1.23 | 0.23 | 1.00 | -1.28 |
| 107 | unknown HC2 | control - QLE | -1.89 | 0.32 | 54 | -5.91 | 0.00 | 0.00 | -3.70 |
| 109 | unknown HC3 | control - methoprene | -0.31 | 0.26 | 54 | -1.21 | 0.23 | 1.00 | -1.24 |
| 110 | unknown HC3 | control - QLE | -1.33 | 0.28 | 54 | -4.71 | 0.00 | 0.00 | -2.51 |
| 112 | unknown HC4 | control - methoprene | 0.04 | 0.24 | 54 | 0.17 | 0.87 | 1.00 | 1.03 |
| 113 | unknown HC4 | control - QLE | -0.45 | 0.26 | 54 | -1.75 | 0.09 | 1.00 | -1.37 |

Table S2: Identifications of egg surface compounds, with measured retention times (RT) and retention indices (RI).

**RT RI identification**

17.88 2103 C21

18.90 2197 C22

19.97 2295 C23

20.37 2332 11-;9-;7-MeC23

20.52 2347 5-MeC23

20.75 2367 3-MeC23

21.04 2394 C24

21.42 2430 12-;10-;8-MeC24

21.65 2452 4-MeC24

21.85 2471 C25:1

22.11 2495 C25

22.48 2530 15-;13-;11-MeC25

22.66 2546 5-MeC25

22.91 2569 3-MeC25

23.29 2603 3,13-;3,11-;3,9-diMeC25

23.59 2630 13-;12-;10-MeC26

23.88 2656 4-MeC26

24.07 2673 C27:1

24.34 2697 C27

24.73 2732 13-;11-;9-MeC27

25.05 2760 11,15-diMeC27

25.20 2773 3-MeC27

25.57 2805 3,11-;3,9-diMeC27

26.21 2862 4-MeC28

26.67 2901 C29

27.04 2934 15-;13-;11-MeC29

27.53 2977 3-MeC29

27.89 3008 C30

28.73 3083 C31:1

28.97 3103 C31

29.32 3134 15-;13-;11-MeC31

29.43 3144 unidentified HC 1

29.66 3164 unidentified HC 2

31.22 3304 C33

31.55 3334 15-;13-MeC33

32.72 3441 unidentified HC 3

33.97 3558 11,15-;13,17-diMeC35

35.76 3724 unidentified HC 4

Table S3: Raw data of egg-policing experiment.

| **Colony** | **treatment** | **WLE_initial** | **policed** | **non_policed** |
| --- | --- | --- | --- | --- |
| Vv1 | methoprene | 180 | 10 | 170 |
| Vv1 | acetone | 23 | 2 | 21 |
| Vv10 | methoprene | 327 | 14 | 313 |
| Vv10 | acetone | 0 | 0 | 0 |
| Vv11 | methoprene | 186 | 16 | 170 |
| Vv11 | acetone | 45 | 15 | 30 |
| Vv12 | methoprene | 288 | 77 | 211 |
| Vv12 | acetone | 0 | 0 | 0 |
| Vv14 | methoprene | 8 | 0 | 8 |
| Vv14 | acetone | 0 | 0 | 0 |
| Vv15 | methoprene | 0 | 0 | 0 |
| Vv15 | acetone | 0 | 0 | 0 |
| Vv16 | methoprene | 240 | 18 | 222 |
| Vv16 | acetone | 0 | 0 | 0 |
| Vv17 | methoprene | 5 | 4 | 1 |
| Vv17 | acetone | 28 | 12 | 16 |
| Vv3 | methoprene | 33 | 11 | 22 |
| Vv3 | acetone | 22 | 13 | 9 |
| Vv4 | methoprene | 260 | 6 | 254 |
| Vv4 | acetone | 128 | 6 | 122 |
| Vv6 | methoprene | 434 | 194 | 240 |
| Vv6 | acetone | 86 | 68 | 18 |
| Vv8 | methoprene | 443 | 96 | 347 |
| Vv8 | acetone | 59 | 38 | 21 |
| Vv9 | methoprene | 346 | 17 | 329 |
| Vv9 | acetone | 90 | 5 | 85 |

Table S4: Chemical data of the worker-laid eggs.

|  | acetone (n=14) | | methoprene (n=26) | | queens (n=17) | |
| --- | --- | --- | --- | --- | --- | --- |
|  | average | sd | average | sd | average | sd |
| **C21** | 0.20 | 0.10 | 0.20 | 0.13 | 0.49 | 0.25 |
| **C22** | 0.23 | 0.11 | 0.21 | 0.10 | 0.38 | 0.25 |
| **C23** | 4.45 | 1.18 | 2.61 | 0.94 | 2.57 | 0.77 |
| **11-;9-;7-MeC23** | 0.94 | 0.33 | 0.49 | 0.24 | 0.71 | 0.23 |
| **5-MeC23** | 0.53 | 0.16 | 0.34 | 0.15 | 0.55 | 0.20 |
| **3-MeC23** | 1.99 | 0.77 | 1.02 | 0.44 | 0.79 | 0.23 |
| **C24** | 4.18 | 1.37 | 2.14 | 0.78 | 2.39 | 0.88 |
| **12-;10-;8-MeC24** | 1.21 | 0.33 | 0.75 | 0.32 | 0.95 | 0.38 |
| **4-MeC24** | 0.73 | 0.22 | 0.44 | 0.18 | 0.46 | 0.18 |
| **C25:1** | 0.33 | 0.23 | 0.24 | 0.11 | 0.37 | 0.14 |
| **C25** | 21.16 | 4.48 | 14.48 | 3.74 | 15.87 | 5.15 |
| **15-;13-;11-MeC25** | 8.73 | 2.26 | 7.31 | 2.27 | 8.71 | 7.62 |
| **5-MeC25** | 1.92 | 0.54 | 1.76 | 0.55 | 2.05 | 1.92 |
| **3-MeC25** | 6.76 | 1.61 | 6.66 | 2.02 | 5.50 | 2.02 |
| **3,13-;3,11;3,9-diMeC25** | 6.27 | 1.13 | 5.09 | 1.15 | 4.73 | 1.48 |
| **13-;12-;10-MeC26** | 1.39 | 0.26 | 1.21 | 0.29 | 1.38 | 1.18 |
| **4-MeC26** | 0.42 | 0.08 | 0.39 | 0.08 | 0.58 | 0.33 |
| **C27:1** | 0.31 | 0.16 | 0.40 | 0.20 | 0.74 | 0.30 |
| **C27** | 13.86 | 5.85 | 20.86 | 4.24 | 13.86 | 5.48 |
| **13-;11-;9-MeC27** | 4.29 | 1.51 | 4.98 | 1.00 | 6.53 | 5.75 |
| **11,15-diMeC27** | 0.42 | 0.31 | 0.34 | 0.09 | 0.55 | 0.45 |
| **3-MeC27** | 2.68 | 0.41 | 5.01 | 1.52 | 6.42 | 2.16 |
| **3,11-;3,9-diMeC27** | 2.89 | 0.47 | 3.73 | 0.56 | 2.97 | 0.65 |
| **4-MeC28** | 0.49 | 0.21 | 0.49 | 0.22 | 0.80 | 0.30 |
| **C29** | 7.39 | 4.30 | 11.19 | 6.84 | 7.23 | 5.84 |
| **15-;13-;11-MeC29** | 1.35 | 0.35 | 1.90 | 0.67 | 1.73 | 0.57 |
| **3-MeC29** | 0.75 | 0.33 | 1.28 | 0.64 | 2.86 | 0.96 |
| **C30** | 0.71 | 0.22 | 0.94 | 0.33 | 1.01 | 0.25 |
| **C31:1** | 0.25 | 0.10 | 0.26 | 0.08 | 0.48 | 0.18 |
| **C31** | 0.66 | 0.51 | 0.34 | 0.23 | 1.25 | 1.14 |
| **15-,13-,11-MeC31** | 0.34 | 0.18 | 0.31 | 0.16 | 0.82 | 0.47 |
| **unknown HC1** | 0.47 | 0.40 | 0.76 | 0.63 | 0.78 | 0.34 |
| **unknown HC2** | 0.37 | 0.22 | 0.51 | 0.36 | 1.27 | 0.51 |
| **C33** | 0.32 | 0.06 | 0.29 | 0.08 | 0.42 | 0.26 |
| **15-;13-MeC33** | 0.32 | 0.16 | 0.28 | 0.08 | 0.49 | 0.27 |
| **unknown HC3** | 0.28 | 0.18 | 0.33 | 0.25 | 0.69 | 0.40 |
| **11,15-;13,17-diMeC35** | 0.20 | 0.06 | 0.22 | 0.10 | 0.30 | 0.16 |
| **unknown HC4** | 0.24 | 0.12 | 0.22 | 0.08 | 0.34 | 0.32 |

Table 5: Total surface area of the nests collected. To measure the surface area, the combs were separated and placed it flat on a table. A ruler was used as scale to be photographed and the area of the combs was estimated using ImageJ (version 1.53e).

|  | **surface area (cm^2^)** |
| --- | --- |
| Vv1 | 1028.821 |
| Vv3 | 878.668 |
| Vv4 | 2503.312 |
| Vv6 | 1446.92 |
| Vv8 | 4368.451 |
| Vv9 | 2335.968 |
| Vv10 | 2044.752 |
| Vv11 | 1392.724 |
| Vv12 | 950.731 |
| Vv14 | 684.266 |
| Vv15 | 1247.166 |
| Vv17 | 1094.998 |
| Vv16 | 1248.191 |
